# Overview of Slovenian Control Programmes for Selected Cattle Diseases, Listed Under Category C, D or E of the European Animal Health Law

**DOI:** 10.3389/fvets.2021.674515

**Published:** 2021-07-09

**Authors:** Jaka Jakob Hodnik, Tanja Knific, Jože Starič, Ivan Toplak, Matjaž Ocepek, Peter Hostnik, Jožica Ježek

**Affiliations:** ^1^Clinic for Reproduction and Large Animals—Section for Ruminants, Veterinary Faculty, University of Ljubljana, Ljubljana, Slovenia; ^2^Institute of Food Safety, Feed and Environment, Veterinary Faculty, University of Ljubljana, Ljubljana, Slovenia; ^3^Department of Virology, Institute of Microbiology and Parasitology, Veterinary Faculty, University of Ljubljana, Ljubljana, Slovenia; ^4^National Veterinary Institute, Veterinary Faculty, University of Ljubljana, Ljubljana, Slovenia

**Keywords:** bovine, disease control, legislation, cattle trade, disease surveillance, infectious diseases

## Abstract

The European Union (EU) regulates the control of cattle diseases listed in categories A and B of the European Animal Health Law (AHL). However, no strict mandatory EU regulation exists for the control of other cattle diseases that are listed in categories C, D and E. Slovenia has five control programmes (CPs) for the latter cattle diseases: bovine viral diarrhoea (BVD), infectious bovine rhinotracheitis (IBR), enzootic bovine leukosis (EBL), bluetongue and anthrax. Two (IBR and BVD) are voluntary and the others (EBL, anthrax and bluetongue) are compulsory. The three compulsory CPs are funded by the government. All the CPs are run by the government and laboratory tests are performed by the National Veterinary Institute. The rules for the CPs are laid down in Slovenian legislation. In addition, there is a national directive for the control of salmonellosis. Both BVD and IBR are endemic and have CPs based on increased biosecurity, testing and culling or vaccination, financed by the animal owners. Slovenia has been officially free of EBL since 2005 and carries out surveillance based on serological testing of a representative number of herds and inspection of carcasses at slaughter or necropsy. Vaccination is the main disease control measure for anthrax (sporadic) and bluetongue (currently perceived free—vaccination since 2017). Lack of motivation of farmers to participate in voluntary disease CPs and to implement and follow strict biosecurity measures are the most pressing issues in improving the health status of Slovenian cattle. An overview of the existing CPs and the circumstances leading to their implementation are presented.

## Introduction

A list of 24 diseases listed under category C, D, or E of the new European Animal Health Law (AHL) [(EU) 2016/429] controlled in at least one member country has recently been compiled (2020) as part of the European Union (EU) COST action SOUND control (CA17110) (1). Each member country was encouraged to write a summary of the CPs for these diseases in their country and to indicate the disease status for the remaining diseases.

In Slovenia there are five control programmes (CPs) in place for infectious cattle diseases with a lower categorization in the AHL (C, D, or E) and a directive for controlling *Salmonella* spp. outbreaks on farms. The CPs are designed to take account of the specific cattle rearing situation in Slovenia (communal alpine pastures, lack of fattening calves, the close proximity of farms, and small herds) and the geographical conditions. All the programmes are implemented by the government and incorporated into the Slovenian legislation. The National Veterinary Institute (NVI), which is part of the Veterinary Faculty, performs all the diagnostic testing for the CPs. Sampling and vaccination in the CPs are carried out by private veterinary practises authorised by the Administration for Food Safety, Veterinary Sector and Plant Protection (AFSVSPP). Bovine viral diarrhoea (BVD) and infectious bovine rhinotracheitis (IBR) have been endemic for several decades (2, 3) and voluntary CPs based on testing and culling or vaccination have been in place since 2014 and are funded by the animal owners. Slovenia has been officially free of enzootic bovine leukosis (EBL) since 2005. Compulsory vaccination is the main strategy to control anthrax and bluetongue (BT). Slovenia has a sporadic occurrence of anthrax and is currently perceived free from BT. The CPs for EBL, anthrax, and BT are compulsory and are funded by the government.

This paper reviews the structure of the Slovenian cattle industry, the details of the existing CPs and provides the status for the other cattle diseases in categories C, D, or E of the AHL for which CPs are in place within Europe.

## Overview of the Cattle Production in Slovenia

**Figure 1:**
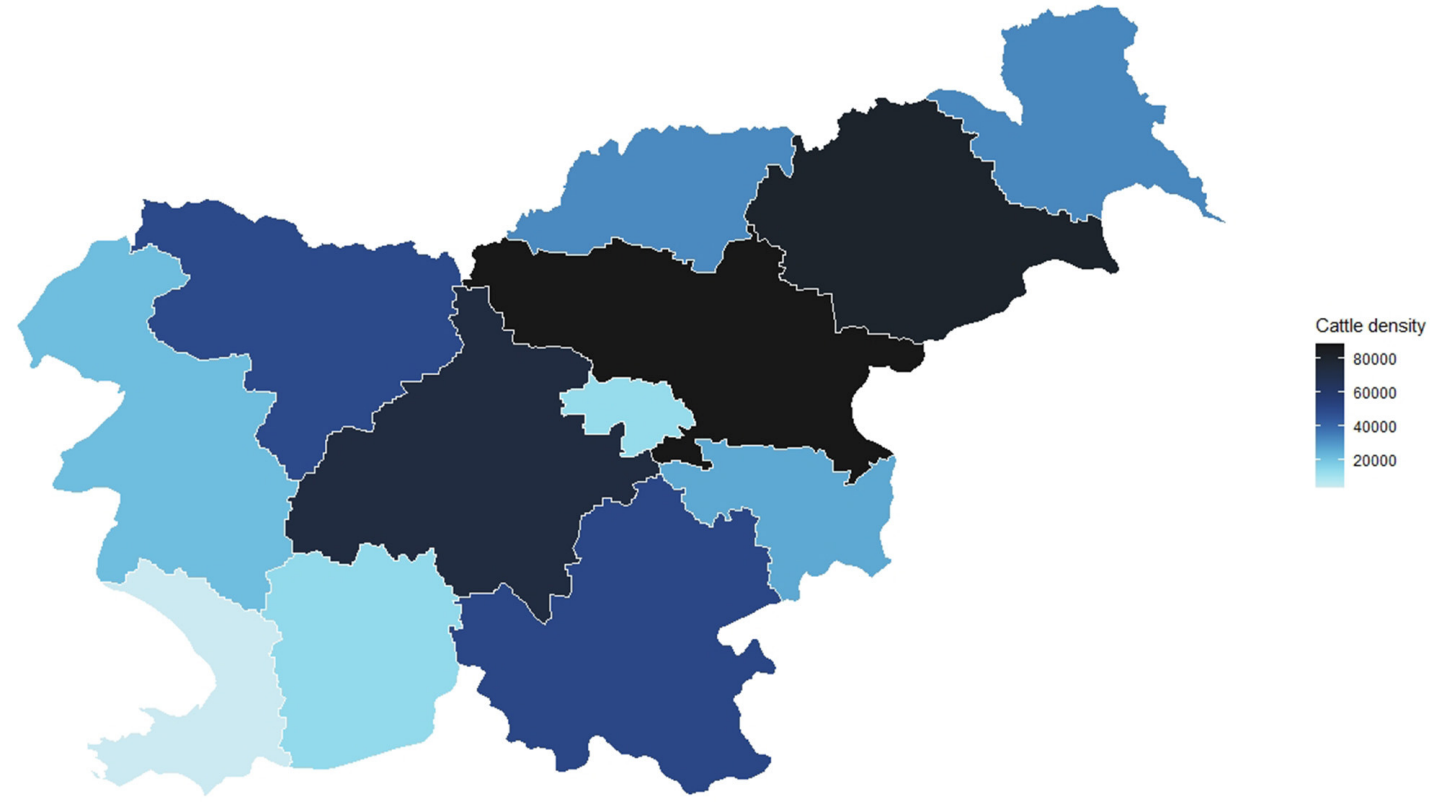
Cattle density in Slovenia by statistical regions.

**Figure 2:**
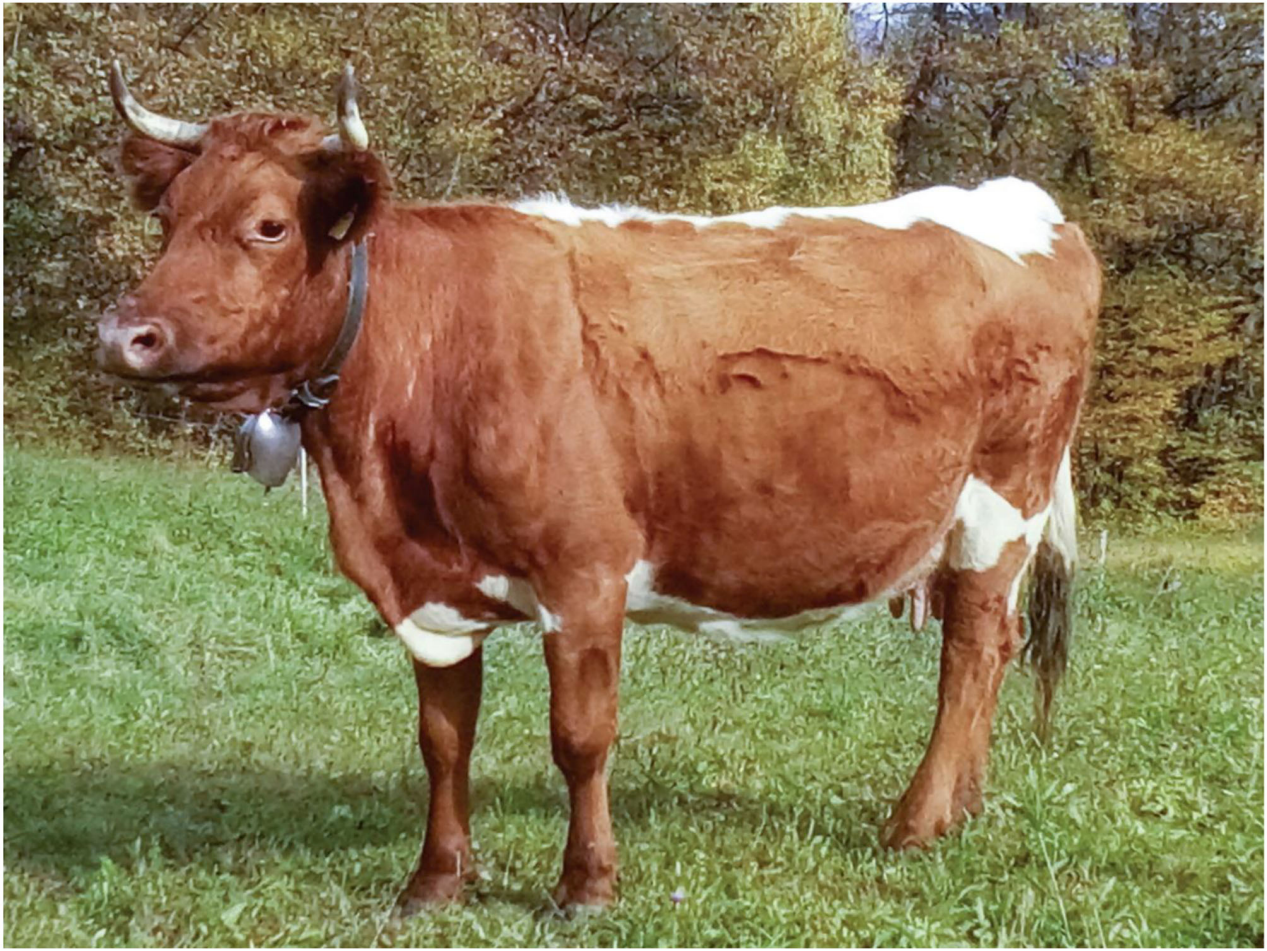
Autochthones Slovenian Cika breed [Foto by Podobnik Franci https://www.cikastogovedo.si/wp-content/uploads/2018/02/veselka-si-43459306-podobnik-franci.jpg (accessed December 21, 2020)].

Slovenia is a small country located in Central Europe south of the Alps. Cattle production is one of the most important agricultural sectors, with about 0.5 million animals. In Slovenia, most cattle herds are family owned and relatively small (4). All cattle holdings and cattle in Slovenia have to be registered at the AFSVSPP. The structure and characteristics of the Slovenian cattle population in 2019 are presented in Table 1 and Figure 1. At the end of 2019, the Simmental breed was the most numerous followed by Holstein and Brown Swiss, while 1% of the cattle population was represented by the autochthonous Cika breed (Figure 2). The rest of the animals (46%) were either Limousin, Charolais, crossbreeds or animals where the pedigree was unknown (4). The number of holdings with cattle decreased from 30,351 in 2018 to 29,615 in 2019 while the number of animals per holding increased from 15.2 in 2018 to 15.8 in 2019 (4, 6). Smaller family farms tend to be more diverse in the animal species that they rear on the farm, compared to bigger enterprise holdings, which rear exclusively cattle (7). In 2019, the density of cattle in Slovenia was 23 cattle per km^2^ (8).

**Table 1:** The structure and characteristics of the Slovenian cattle population in 2019.

Number of cattle	466,911
Number of cattle herds	29,615
**Average herd size**	
- Dairy	17.5
- Non-dairy	3.7
- Total	16.8
**Ownership**	
- Family owned	98.3%
- Agricultural enterprises	1.7%
**Cattle system**	
- Dairy	19%
- Non-dairy	81%
**Animal structure**	
- Cows	34%
- Calves	29.8%
- Heifers	20.8%
- Bulls	16.8%
**Breeds**	
- Simmental	29.9%
- Holstein	16.8%
- Brown Swiss	4.4%
- Cika	0.9%
- Others (Limousin, Charolais, crossbreeds, …)	46.3%
**Average production parameters**	
- Milk yield	7,043 kg
- Simmental breed	5,890 kg
- Holstein	8,261 kg
- Insemination index	1.92
- Calving interval	422 days (dairy cows)/438 days (beef cows)
- Days open	138 days
- Daily gain in calves	1,096 g/day (0–210 days)

*The information is summarised after (4, 5)*.

Cattle are mostly reared indoors, but in Mediterranean, Alpine and pre-Alpine regions cattle have access to pastures for several months. The duration of grazing depends on the region.

## Factors Affecting Cattle Disease Control in Slovenia

In Slovenia, the supply of beef calves does not meet demand. Therefore, farmers import calves from Middle and Eastern European countries. In the period between 2010 and 2016, most of the calves were imported from the Czech Republic (58.2%), followed by Hungary (10.2%), Romania (9.5%), and Slovakia (9.2%) (9). Imported calves are usually cheaper than those originating from Slovenia. The health status of imported calves is not checked for diseases in categories C, D, or E of the AHL and quarantine is not carried out before they are introduced into the herds, as it is not mandatory. This is one of the reasons why beef farmers are less inclined to the national eradication of IBR and BVD as it would lead to quarantine restrictions and laboratory testing of imported animals from non-free countries, resulting in additional costs. Furthermore, these diseases are not perceived as a major problem by many beef farmers, although beef cattle herds with infectious respiratory disease outbreaks are observed each year (10). In 2019, Slovenia exported 37,177 cattle and imported 32,177 cattle (8).

Beside the unwillingness of beef importers to support a systemic approach to improve the health status of cattle, there are also other factors, characteristic for Slovenia, that would need to be addressed. In the Alpine region, many farms use communal mountain pastures, which pose a risk of disease transmission between herds. As arable land is limited, farms in most regions are located close to each other, with boundaries being separated by only a single fence line. Dairy farms face low milk prices limiting opportunities for investment. The size of farms and the level of production is also affected by the fact, that about 75% of the available Slovenian agricultural land is located in areas less favourable for agriculture, 56% of which is on steeply sloped terrain (11). These factors make Slovenian cattle farmers less economically competitive compared to farmers from countries with more favourable farming conditions. The lack of financial reward has probably driven a lack of younger people engaging in cattle production leading to an ageing population of farmers. Most farm owners are over 55 years old [57 years on average in 2016 (12)] and are likely to be less open to change and investment (13).

Farmers who have achieved eradication of a particular disease on their farm or have a favourable herd health status have already implemented biosecurity measures such as foot disinfection barriers and a change of clothes for visitors. However, the study in 2021 found that the majority of farms do not consider biosecurity as a top priority and buy animals with unknown health status and often share equipment with their neighbours (14).

In Slovenia, all traded cattle must be free of brucellosis, tuberculosis and EBL. Animals that are traded must comply with the guidelines prescribed in Commission Regulation (EC) No. 1266/2007 of 26 October 2007 on implementing rules for Council Directive 2000/75/EC regarding the control, monitoring, surveillance, and restrictions on movements of certain animals of susceptible species in relation to bluetongue. Since 1997 all young bull stations and insemination centres in Slovenia have been free of brucellosis, tuberculosis, EBL, BVD, IBR, trichomonosis, bovine genital campylobacteriosis, and BT. All introductions of animals into young bull stations and insemination centres are under strict and regular veterinary control^1^

For larger cattle shows animals must be tested for IBR and BVD prior to the show, while for smaller shows the rules are not so strict unless a farmer wishes to maintain his BVD or IBR status, in which case cattle from negative herds must be kept separate.

## Economic Losses Due To Diseases for Which CPS are In Place

Losses due to livestock diseases are divided into direct losses due to the impact of the disease on production and life span and indirect losses resulting from expenditure on disease control and prevention and lost revenue (15). Although there are no detailed studies on disease losses in Slovenia, we can consider their economic importance based on studies conducted in other countries. BVD is associated with large economic losses, either directly through reduced productive performance in cattle herds or indirectly, such as expenditure on CPs (16). In the case of BVD, several studies have shown that CPs are economically justified (17). The economic significance of losses associated with IBR is not yet clear due to lack of data. However, there is evidence that it causes production losses due to respiratory disease, reduced fertility, abortions and reduced milk yield (18). Production losses due to EBL are controversial, but even in studies showing losses, they appear to be low (19). Economic importance results mainly from trade bans (15). In the case of BT, production losses vary from relatively low in endemic situations to substantial losses in epidemic situations. Losses are caused by reduced fertility, mortality of older animals and reduced milk production. Most of the costs associated with BT are the result of prevention and control measures (vaccination, restrictions on animal movements, impact on markets), the magnitude of which appears to be far greater than direct disease losses (20). As a zoonosis, anthrax poses a public health risk. Although production losses due to anthrax are estimated to be low (few dead animals), overall losses due to indirect losses may be significant (21).

## Description of Existing Control Programmes in Slovenia for Selected Cattle Diseases

### Bovine Viral Diarrhoea (BVD)

**Figure 3:**
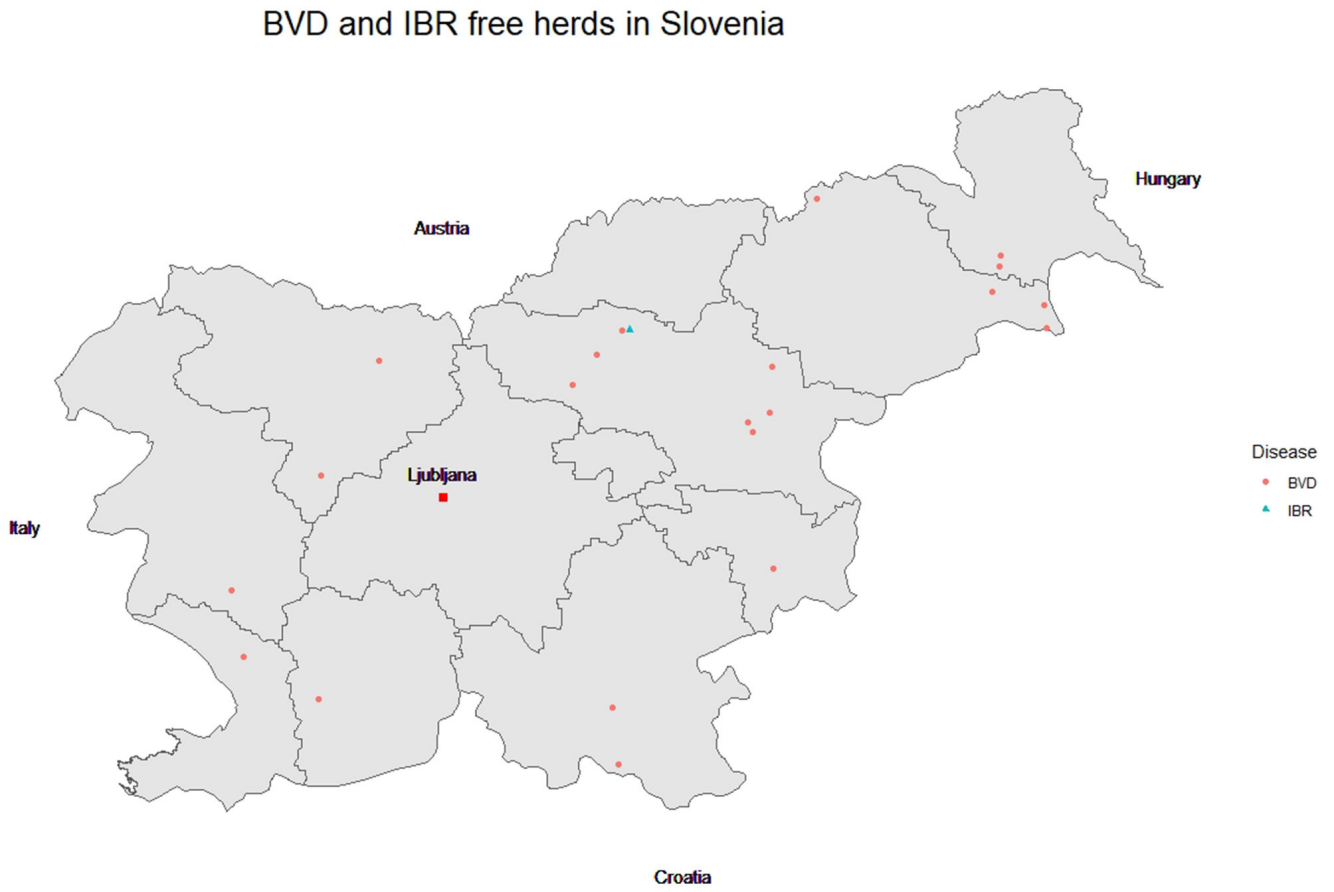
Location of bovine viral diarrhoea (BVD) or infectious bovine rhinotracheitis (IBR) officially free herds. Note that the only herd free of IBR also has a BVD-free status but the markers are misaligned for better visualisation.

In Slovenia, cattle owners have been able to acquire BVD-free herd status since 2014 (22), and currently 21 herds have this status (Figure 3). The CP is implemented on a national level, is voluntary and is financed by herd owners. The BVD-free status is awarded on a herd level. The programme is a modification of the successful BVD eradication programme first implemented in Sweden (23). The programme follows a prescribed rule which sets out the conditions for recognition, acquisition and maintenance of a BVD-free herd status (24). The last systemic surveillance for BVD prevalence was conducted in 2003. At that time, 12,885 breeding animals from 307 holdings were serologically (ELISA) tested, and 16.8% of animals and 50.2% of holdings were BVD positive (25).

Owners can apply for BVD-free herd status at the regional office of the AFSVSPP. If all conditions are met, the herd will be granted BVD-free status. All herds that have been granted the status are listed on the AFSVSPP website^2^

The conditions for BVD-free status are; No confirmed case of BVD virus (BVDV) infection on the holding in the last 12 months, all cattle on the holding are free from clinical signs indicative of BVDV infection. The herd is kept separate from other herds that have a lower BVD health status (direct contact between animals is not permitted), only cattle from BVD-free herds are included into the herd, alternatively cattle are quarantined, and tested for evidence of infection (ELISA and RT-PCR), and female cattle are only inseminated with semen from bulls or serviced by bulls that are proven to be BVDV-free. In addition, the herd must have two consecutive negative serological tests (with antibody ELISA) of cattle aged 7–13 months (“spot test”), at least 6 months apart. If there is no animal in this age group on the holding, animals in the 14–21-month age group will be tested. Vaccination is not allowed in herds participating in the CP.

If the owner wishes to achieve BVD-free herd status, but the initial or subsequent serological test is positive, the herd must eradicate the disease and retest 7–13-month-old calves serologically twice at least 6 months apart. A possible eradication plan is suggested in the Rule: all animals in the herd must be tested for the presence of BVDV in the blood using RT-PCR [identification of Persistently Infected (PI) animals] and all positive animals must be culled. All new-born calves born in the year following the removal of the last positive animal must be tested for BVDV in the first week of life; positive animals should be culled from the herd as soon as possible. One year after the last PI has been removed from the herd, serological testing of cattle aged 7–13 months is required. If the results of all tested animals are negative, the spot test is repeated after 6 months; if the results of these tests are negative and the preventive measures for BVD-free status are fulfilled, the herd can apply for BVD-free status. If the results of the tests are not negative, the above measures are continued.

BVD free status is granted for a period of 1 year. To maintain the status, a herd must be “spot tested” annually. In addition, the owner must ensure that the local veterinarian investigates compliance with the conditions for BVD-free status. If a herd no longer complies with the conditions for maintaining BVD-free status, the status is lost (22).

The status is temporarily lost if only one animal tests positive for antibodies in the spot test. The status is renewed when the serologically positive animal is culled and, after 30 days, all animals between 9 and 15 months of age test negative for BVDV antibodies. If no animal in this age group is present on the farm, animals between 16 and 23 months of age are tested.

Testing of young stock was chosen for initial testing because they are more likely to be exposed to PIs, which increases the likelihood of detecting BVDV infection in the herd. Since almost half of the cattle herds in Slovenia have a low prevalence of BVDV, this provide the opportunity to apply for BVD-free status within 6 months. Due to the proximity of cattle holdings in Slovenia and the fact that about 15% of cattle herds have PIs (26), the risk of reintroduction of the virus is quite high, so many farmers are reluctant to participate in the eradication programme. Maintaining the status also entails additional costs for laboratory testing and restrictions on the purchase of new animals, but farmers do not receive any additional privileges or rewards for having a BVD-free herd status (except being listed on the AFSVSPP website). However, many farmers who are aware of the loses associated with BVD have successfully eradicated BVD and thus have a favourable health status, although because they do not participate in the official programme, they cannot have the official status.

### Infectious Bovine Rhinotracheitis (IBR)/Infectious Pustular Vulvovaginitis (IPV)

The last extensive serological survey for IBR was performed in 2006. Animals older than 24 months (204,662 cattle), from 35,991 farms were serologically tested by ELISA. Positive animals were found in 1,287 farms (3.6%) (27).

In Slovenia, a voluntary national eradication programme has been in place since 2015. The CP is described in the Rule prescribing the conditions for recognition, acquisition and maintenance of a IBR-free herd status (28). All costs for acquiring and maintaining the status are funded by the owners. In this programme, an animal is considered infected if virus can be detected or the animal is seropositive for antibodies to the entire Bovine alphaherpesvirus-1 (BoHV1).

A holding keeping bovine animals is considered free of BoHV1 infection if it meets the following conditions of the CP; no suspicion of BoHV1 infection has been detected on the holding in the last 6 months, all cattle on the holding are free from clinical signs indicative for BoHV1 infection, the herd must be separated from herds that have a lower IBR health status at all times (direct contact between animals of different health statuses is prevented), only cattle from IBR-free herds or quarantined and negatively tested cattle may be introduced into the herd, cows and heifers are serviced or inseminated with semen from IBR-free bulls, and the herd has been serologically tested twice in an interval of 5–7 months with negative results. The sampling protocol is prescribed in Annex 3 of Commission Decision 2004/558/EC.

The owner must submit an application for IBR-free herd status to the regional office of the AFSVSPP. If all requirements are met, the herd is granted IBR-free status. All herds that have been granted the status are listed on the AFSVSPP website^3^

If a herd does not meet the above conditions, the owners must contact their local veterinarian who will prepare an eradication plan. The eradication plan most commonly implemented in Slovenia consists of identification and culling of infected animals or vaccination of animals with a marker vaccine until the last wild type IBR virus antibody-positive animal is culled. The former is recommended if <10% of the animals in the herd are positive at serological testing (29). When all requirements are met, the owner may apply for the free status.

To maintain the status, the owner must comply with the conditions to obtain free status, except for the initial testing, but must perform annual serological testing with negative results. The sampling protocol is prescribed in Annex 3 of Commission Decision 2004/558/EC. In addition, the owner must ensure that the local veterinarian confirms that the herd is compliant with the Rule each year. If the herd no longer complies, the status will be lost. Regardless, if only one animal tests positive in the annual serological testing the status is temporarily lost until the positive animal is culled and others are serologically tested negative twice.

Currently, only one herd has IBR-free status (Figure 3). Slovenian insemination centres have been IBR negative since 1975 with one minor outbreak in one centre that was quickly brought under control (30). The insemination centres in Slovenia also adhere to this CP to maintain their status. Farmers are not very motivated to participate in the CP as it involves additional costs and they cannotintroduce animals into the herd without implementing quarantine measures. Free herds do not receive any privileged status compared to positive herds. Based on the last serological screening, the prevalence of IBR in Slovenia is low. Positive herds belong predominantly to the Holstein breed, which is most likely the result of the closure of state collective farms which had a high prevalence of IBR (in the former Yugoslavia) and the auctioning-off of their animals. Herds of other breeds in Slovenia are rarely infected (29).

### Enzootic Bovine Leukosis (EBL)

Slovenia has a surveillance programme to prove national EBL-free status. If EBL-positive animals are found, eradication measures follow. All sampling and testing are paid by the government, all other costs are the responsibility of the owner. Slovenia has been granted official EBL-free status by the EU (<0.2% infected herds) in 2005. The last reported case found by active surveillance was in 2006, and since then there have been eight cases in imported cattle in Slovenia (31).

In order to maintain the national officially free status, Slovenia has an active surveillance programme that includes the serological testing of cattle older than 12 months. The number of animals and herds to be tested annually is determined by the AFSVSPP. All positive and suspect cases are confirmed by retesting with serological and molecular methods. Passive surveillance is carried out in slaughterhouses during post-mortem examination of carcasses, where samples of all carcasses with tumour-like lesions are examined for EBL. The same procedure is used when tumour-like lesions are found at necropsy. Passive surveillance is also carried out in the field, where veterinarians must report animals with enlarged lymph nodes, ill-thrift or marked lymphocytosis with lymphocytes comprising more than 65% of the white blood cells. The official veterinarian must then carry out an epidemiological investigation and ensure the serological testing of all animals on the holding. All movement of animals other than for slaughter is prohibited, all animals suspected of being infected must be isolated, and disinfection barriers must be placed at the entrance to the holding and pens. If EBL is confirmed, by serological or molecular tests or at post-mortem examination, all positive animals and any potentially infected offspring of infected dams must be culled within 30 days after the owner and the official veterinarian have been informed of the test results. All movement of animal products from the farm is prohibited. Cleansing and disinfection must be carried out by a registered organisation^4^ The herd regains the status when all positive animals are culled and all other animals older than 12 months are tested twice, 3 months after the removal of the last positive animal and 4–12 months after. All tests must be negative.

### Bluetongue

The last reported case of bluetongue caused by serotype 4 was in 2016 (OIE report 2018). The national, compulsory vaccination and surveillance programme was launched in 2017 and is funded by the government.

The Slovenian CP is based on Council Directive 2000/75/EC of 20 November 2000 laying down specific provisions for the control and eradication of bluetongue and Commission Regulation (EC) No. 1266/2007 of 26 October 2007 on implementing rules for Council Directive 2000/75/EC as regards the control, monitoring, surveillance and restrictions on movements of certain animals of susceptible species in relation to bluetongue.

All bovines and small ruminants must be vaccinated every year. Cattle and goats are initially vaccinated twice 3 weeks apart, then once a year. Sheep are vaccinated once a year. All animals must be vaccinated during the vector-free season (usually from January to April). Inactivated bluetongue serotype 4 vaccines are used. In 2018, 435,246 cattle were vaccinated in Slovenia (31). Some animals (selected by AFSVSPP) are left unvaccinated to serve as sentinels and are serologically tested twice (before April and in December). To confirm the disease, all positive animals are retested and if they are not negative, they are resampled and retested using serological and molecular methods. Entomological surveillance for *Culicoides* spp. is also conducted. Samples are collected every week in winter and every other week in summer in 10 locations across the country using insect traps. The results are used to monitor the number of *Culicoides* spp. and the duration of the vector-free season throughout the year. The costs of vaccination and testing are covered by the government^5^ Owners are compensated for culled animals.

Some owners are reluctant to vaccinate their animals because the modified live bluetongue vaccine used in some countries outside the EU has been associated with abortions and clinical disease (32, 33).

### Anthrax

The latest version of the anthrax CP was put in force in 2016^6^ The programme is compulsory, national, and financed by the government. In Slovenia, the last recorded case of anthrax was on 21st August 2015^7^

Suspicion of anthrax is based on clinical signs or post-mortem examination. The veterinarian reporting the suspicion takes blood samples from live animals or sends carcasses for laboratory diagnosis, informs the regional office of the AFSVSPP and gives additional instructions to the owner to prevent the spread of infection. The diagnosis is confirmed at the NVI with a pathomorphological examination, bacteriological examination and real-time PCR. The official veterinarian conducts an epidemiological investigation and puts the following measures into force: (1) no movement of animals or their products, (2) euthanasia of all animals that do not test negative at diagnostic testing, (3) no slaughter or opening of carcasses, (4) vaccination of all ruminants and equids, (5) destruction of carcases of dead animals, (6) destruction and disinfection of animal waste material, cleaning and disinfection of all equipment which has been in contact with the infectious material, (7) disinfection of the ground where animals died, (8) pest control (of insects and rodents) and (9) other measures to sanitise the holding.

Regardless of the movement ban, animals showing no clinical signs after 21 days (longest incubation period) may be slaughtered with the approval of the official veterinarian. In addition, milk from clinically healthy animals may be used for human consumption if it is heat treated (at least to pasteurisation temperature) in approved facilities under official control. At-risk animals may be treated with antibiotics. Treated animals must be vaccinated 10 days after the end of antibiotic treatment, as a live attenuated vaccine is used. Disinfection and pest control must be carried out by a registered organisation.

**Figure 4:**
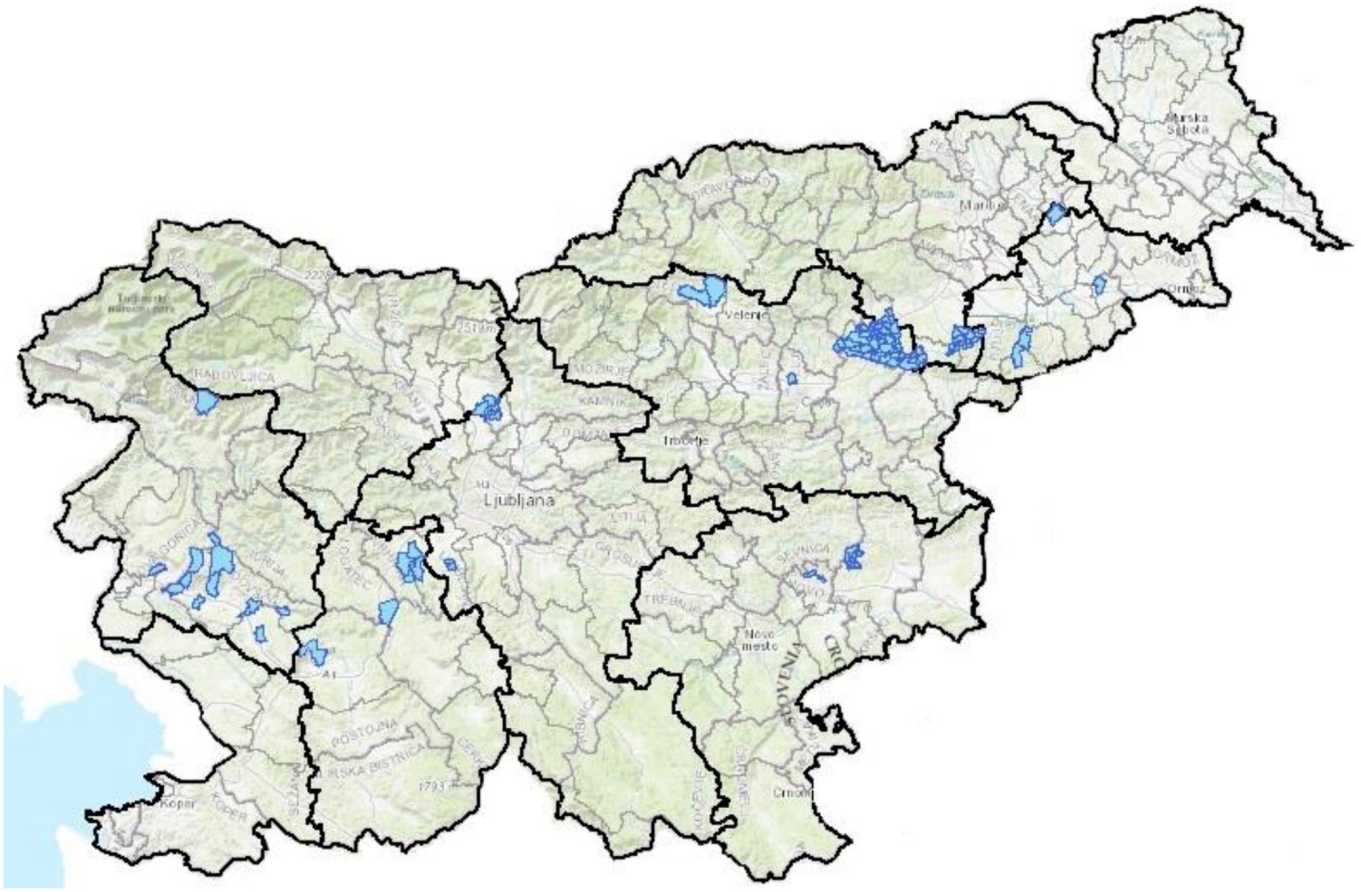
Location of the all 106 anthrax districts in Slovenia, in 2019 (34).

When the disease is confirmed, the AFSVSPP establishes an anthrax district and makes the information publicly available on its website. There are currently 106 anthrax districts in Slovenia (Figure 4). All ruminants and equids must be vaccinated 3 weeks before the start of the grazing season in the district or receiving feed from an anthrax district. All measures on the affected holding are in effect for 21 days from the date all measures and disinfection have taken place. Vaccination measures in an anthrax district are in force for 50 years. In 2018, 16,449 cattle, 138 equids and 970 small ruminants were vaccinated in Slovenia. Vaccination is not associated with any additional restrictions (31).

### Salmonellosis

The directive for the control of salmonellosis has been in force since 1999. Sporadic cases still occur in Slovenia. Control measures are described in the Directive for detection, prevention and eradication of salmonellosis (35). The measures are compulsory and the costs are borne by the owner.

Suspicion is based on clinical signs (diarrhoea, abortion storm, and death) or when salmonellosis is confirmed in other species on the farm. A local veterinary organisation must take rectal swabs, samples of bedding and feed, and submit dead animals for necropsy. They must also instruct the owner on measures to prevent the spread of the disease (prohibit movement of animals, restrict movement of people, and implement additional sanitary measures) and order the destruction of infected carcasses. The disease is confirmed by bacteriological examination. If salmonellosis is confirmed, the official veterinarian must order the disinfection of feed, treatment of animals with antibiotics on the basis of an antibiogram, disinfection, pest control and other sanitary measures. The measures may be stopped when two bacteriological tests on rectal swabs from all animals in infected management groups, performed 7 and 14 days after the end of the treatment are negative.

Because salmonellosis has similar clinical signs to other diarrhoeal diseases, samples are rarely collected for laboratory diagnosis and animals are often treated symptomatically. The annual number of reported human *Salmonella* spp. cases in Slovenia ranged between 253 and 615 (median = 366) from 2009 to 2018. Most outbreaks were the result of consumption of undercooked chicken meat or eggs (36).

### Epidemiological Situation for Other Cattle Diseases Listed Under Category C, D, or E in the Animal Health Law

A COST action SOUND control is researching the cattle diseases listed under category C, D, or E in the AHL for which CPs exist in European countries. The action has compiled a list of 24 diseases that are controlled in at least one country (1). The Slovenian status for these diseases not already mentioned in the text is shown in (Table 2).

**Table 2:** Disease status for Slovenia of cattle diseases listed under category C, D, or E in the Animal Health Law for which CPs exist in European countries.

**Disease**	**Disease status**	**Reference**
Bovine genital campylobacteriosis	Free	(31)
Trichomonosis	Free	(31)
Epizootic haemorrhagic disease	Never reported	(31)
Johne's disease	Endemic	(37)
Q fever	Sporadic	(31)
Surra	Never reported	(31)
*Mycoplasma mycoides* subsp. *mycoides*	Never reported	(31)
Leptospirosis	Sporadic	Expert opinion
*Mycoplasma bovis*	Endemic	(10)
Aujeszky's disease	Officially free	(38)
*Streptococcus agalactiae*	Sporadic	Expert opinion
Bovine coronavirus	Endemic	(10)
*Staphylococcus aureus*	Endemic	Expert opinion
Bovine respiratory syncytial virus	Endemic	(10)
Bovine digital dermatitis	Endemic	Expert opinion
*Trichophiton verrucosum*	Endemic	Expert opinion
Liver fluke	Endemic	Expert opinion
Neosporosis	Endemic	Expert opinion

## Discussion

Cattle production is an important part of the Slovenian economy. In 2019, the agricultural sector generated 1.2% (€1.3 billion) of the national gross domestic product (GDP), with the cattle sector accounting for 26% (€158 and €188 million from beef and milk production, respectively) (39, 40).

CPs in Slovenia are designed to take account of the specific cattle rearing situation and the prevalence of these diseases. Most cattle movements are within the country (71.3%), with the remaining 28.7% attributed to import and export. Within Slovenia, there are about 150 thousand cattle movements each year (excluding export and import) (9). Neglecting to check the health status of purchased animals before adding them to the herd or introducing them on to communal pastures facilitates the introduction and spread of infections between animals. A good example of this is the introduction of new strains of BVDV-1 and their local spread through the use of communal pastures (41–43). A study determining the genotype of all the BVDV isolates in Slovenia collected between 1997 and 2001 showed that the most affected regions were Gorenjska and North Primorska (West and North-West of Slovenia), which use Alpine pastures in summer. BVDV-1f was the most frequently isolated genotype (42). An observed prevalence of IBR and BVD is also the result of large state-owned collective farms auctioning-off their cattle when they closed between 1990 and 1995, spreading the infection throughout Slovenia. These as well as other farms had a high prevalence of IBR and BVD because they imported many breeding dairy cattle during the period when Slovenia was part of the former Yugoslavia (29). BVD CPs in Europe are mostly based on bulk milk sampling and spot tests or tissue tagging (44). Due to the relative high prevalence, small herd size and close contact of animals from different herds, the proposed CP for BVD in Slovenia was designed to sample all individual animals in a specific age group to increase the sensitivity of diagnosis and to facilitate early detection of new outbreaks. Furthermore, as the programme is voluntary and only a small number of herds have achieved official BVD-free status, BVD-free herds are at high risk of reinfection from neighbouring herds with an inferior health status as are herds participating in the IBR CP. Which explains the low participation in both programmes (22 herds are BVD-free and 1 herd is IBR-free). Compulsory national eradication programmes will be necessary in order to further address these diseases within Slovenia. Such programmes can be best implemented if they require no or minimal financial contribution from breeders.

The public-private partnership in Slovenia consists of the government (Ministry and AFSVSPP), veterinary services (Veterinary Faculty and NVI), veterinary associations (Slovenian Veterinary Chamber) and breeders' associations. In Slovenia, each of the traditional dairy breeds has its own breeders' association and a common association for beef cattle breeders. The autochthonous breed Cika also has its own breeders‘ association. All CPs in Slovenia are operated by the government, which has created the legal framework to obtain a free status. Diagnostics are performed by certified laboratories (part of the NVI), which have the knowledge and equipment to operate these CPs. Field work (e.g., sampling, vaccinations, and annual herd health checks) is performed by private veterinary practises that have a concession with the AFSVSPP. Although all stakeholders are involved in discussions when new legislation on control of cattle disease is prepared, the number of farms participating in voluntary CPs is still low. So far, no breeders' association has made disease eradication compulsory for its members, and in Slovenia there is no common association of milk processors. Therefore, the decision whether to participate in voluntary CPs is left to the individual farmer. Few farmers have chosen to maintain a BVD or IBR free status. More farmers have used these or similar programmes to eradicate the disease and gained a favourable herd health status. However, the retention of the free status involves additional cost for sampling and testing but provides no additional benefit because the free herds are not privileged or rewarded (except being listed as free on the AFSVSPP website), and the positive herds have no restrictions or penalties. Also, farmers do not consider the health status of the animal as a top priority when buying new animals and most are not willing to pay extra for BVD or IBR negative animals. Therefore, until clear benefits are provided to free herds both statuses will be maintained by just a few farms.

The sub-Mediterranean, sub-alpine and temperate continental climate resulting from Slovenia's geographical location and global warming have facilitated the introduction of some arboviruses, such as Schmallenberg virus and Bluetongue virus. Bluetongue virus serotype 4 has become endemic on the Balkan Peninsula since 2015. Slovenia has also been endemic for the Schmallenberg virus since 2013 (45).

EBL and BT CPs are compulsory and are based on EU directives, with country-specific measures mandated by these directives. There has been some reluctance by owners to vaccinate their animals against bluetongue for fear of abortions and fertility problems. In 2020, there were outbreaks of BT serotype 4 in many countries in the region^8^ However, the vaccination programme has proven effective, as there has had been no confirmed BT outbreak in Slovenia since the programme began. However, animals are only screened for serotype 4 antibodies; therefore, the detection of other serotypes is based only on passive surveillance. As BT seems to be endemic in the region, the continuation of vaccination is justified.

The salmonellosis directive is based on passive surveillance and only controls herd level outbreaks and the zoonotic risk to humans. The limitation of passive surveillance for salmonellosis is that it does not require the investigation of clinical disease and samples for diarrhoea are rarely taken unless severe outbreaks occur. Several countries in Europe, such as Finland, Denmark, Estonia, Norway, the Netherlands, and Sweden have established salmonellosis CPs (46). They collect samples from carcasses, faeces^9^, blood samples, or bulk tank milk (47, 48). Surveillance systems in European countries were mostly established to control the zoonotic risk to humans. In Slovenia, most cases of salmonellosis are the result of consumption of undercooked chicken meat and eggs (36). Therefore, the control of salmonellosis in cattle herds might not have a large impact on public health.

Regarding the number of cattle diseases listed under category C, D, or E in the AHL (investigated by SOUND control) controlled in European countries, Slovenia is below average with five CPs. The average in Europe is eight CPs per country. Diseases controlled in Slovenia are also controlled in most other European countries (1). The disease status for the controlled diseases is similar to the statuses of other countries in the region, with Austria having a favourable status for BVD and IBR, and Italy having regions free of IBR or regions with compulsory CPs (49). The country's status for the diseases that are not controlled in Slovenia (Table 2) are similar to the statuses of the neighbouring countries (46).

The goal for Slovenia is to implement compulsory national programmes for BVD and IBR and become a BVD- and IBR- free country, following the example of the successful eradication of these diseases by other European countries. However, all previous efforts have been stopped by some cattle breeders' associations due to the high cost of testing and restrictions on importing calves. Based on the available literature from other countries, the economic benefits of the implementation of a national BVD eradication programmes vary depending on the disease control measures and the cattle rearing situation (50). Switzerland, which has similar cattle rearing practises as Slovenia has a successful BVD eradication programme that began with tissue tagging and progressed to serological testing. Their programme has been evaluated to be economically beneficial to the cattle industry (51). Many European countries have used or are using a test and cull or test and vaccinate strategy to eradicate IBR with great success (49). Since Slovenia exports many live cattle to Austria and Italy (9), the eradication of both diseases would facilitate export to these countries and increase the value of cattle. Future efforts should be directed towards optimising the Slovenian BVD CP and motivating farmers and the policy makers to implement a national compulsory CP. IBR eradication also seems unlikely without a government initiative. Slovenia could implement both programmes simultaneously and use the same samples for eradication of both diseases, which would reduce the costs.

Anthrax spores are embedded in the soil in some districts of Slovenia and can survive for up to 50 years; therefore, vaccination seems to be the only way to prevent animal losses and protect public health in these districts. Because of the zoonotic potential of *Mycobacterium avium* spp. *paratuberculosis*, there is a government-funded initiative to develop a paratuberculosis CP following the example of other European countries (37). In the last prevalence study for paratuberculosis in 2008, Slovenia had a favourable epidemiological situation. However, this may no longer be the case if no action is taken (52).

## Conclusion

Slovenia has five CPs in force for cattle diseases listed under category C, D, or E in the AHL for which CPs exist in European countries, which are the result of the specific cattle rearing conditions in the country and the wider region. The goal is to achieve eradication or control of all these diseases and add additional CPs for other diseases, which would increase the commercial value of Slovenian cattle, improve production, and animal welfare.

## Author Contributions

All authors contributed to the information gathering, design, writing, and editing of the manuscript.

## Conflict of Interest

The authors declare that the research was conducted in the absence of any commercial or financial relationships that could be construed as a potential conflict of interest.

## Footnotes

^1^http://pisrs.si/Pis.web/pregledPredpisa?id=PRAV6275 (accessed December 21, 2020).

^2^https://www.gov.si/assets/organi-v-sestavi/UVHVVR/Bolezni-zivali/BVD/42_Seznam-cred-prostih-BVD-posodobljen-24.06.2020.pdf (accessed December 21, 2020).

^3^https://www.gov.si/assets/organi-v-sestavi/UVHVVR/Bolezni-zivali/IBR/1_Seznam_cred_prostih_IBR-IPV_posodobljeno31._5._2017.pdf (accessed December 21, 2020).

^4^http://www.pisrs.si/Pis.web/pregledPredpisa?id=PRAV6813 (accessed December 21, 2020).

^5^http://www.pisrs.si/Pis.web/pregledPredpisa?id=ODRE2602 (accessed December 21, 2020).

^6^http://www.pisrs.si/Pis.web/pregledPredpisa?id=PRAV12802 (accessed December 21, 2020).

^7^https://www.oie.int/wahis_2/temp/reports/en_imm_0000018513_20150826_174821.pdf (31) (accessed December 21, 2020).

^8^Bosnia and Herzegovina: https://www.oie.int/wahis_2/temp/reports/en_imm_0000036334_20201030_103224.pdf (accessed January 29, 2021). Serbia: https://www.oie.int/wahis_2/temp/reports/en_imm_0000036233_20201023_180414.pdf (accessed January 29, 2021). Croatia: https://www.oie.int/wahis_2/temp/reports/en_imm_0000036188_20201224_134407.pdf (accessed January 29, 2021). Albania: https://www.oie.int/wahis_2/temp/reports/en_imm_0000036255_20201030_210311.pdf (accessed January 29, 2021). North Macedonia: https://www.oie.int/wahis_2/temp/reports/en_imm_0000035083_20200717_181213.pdf (accessed January 29, 2021).

^9^https://www.riigiteataja.ee/akt/12814159 (accessed January 29, 2021).
