# Corrigendum: Overview of Slovenian Control Programmes for Selected Cattle Diseases, Listed Under Category C, D or E of the European Animal Health Law

**DOI:** 10.3389/fvets.2021.835395

**Published:** 2022-01-12

**Authors:** Jaka Jakob Hodnik, Tanja Knific, Jože Starič, Ivan Toplak, Matjaž Ocepek, Peter Hostnik, Jožica Ježek

**Affiliations:** ^1^Clinic for Reproduction and Large Animals—Section for Ruminants, Veterinary Faculty, University of Ljubljana, Ljubljana, Slovenia; ^2^Institute of Food Safety, Feed and Environment, Veterinary Faculty, University of Ljubljana, Ljubljana, Slovenia; ^3^Department of Virology, Institute of Microbiology and Parasitology, Veterinary Faculty, University of Ljubljana, Ljubljana, Slovenia; ^4^National Veterinary Institute, Veterinary Faculty, University of Ljubljana, Ljubljana, Slovenia

**Keywords:** bovine, disease control, legislation, cattle trade, disease surveillance, infectious diseases

In the original article, we used the phrase “non-regulated” for cattle diseases that are in fact listed in the New Animal Health Law that went into force in 2021. The corrected **Title, Abstract**, paragraph one, **Introduction**, paragraph one, two and three, header title and paragraph one of **Section two** and **Section three**, (Table 2) caption, **Discussion**, paragraph six, and **Conclusion** appear below.

**Title:**

“Overview of Slovenian Control Programmes for Selected Cattle Diseases, Listed Under Category C, D or E of the European Animal Health Law”

**Abstract, paragraph one:**

The European Union (EU) regulates the control of cattle diseases listed in categories A and B of the European Animal Health Law (AHL). However, no strict mandatory EU regulation exists for the control of other cattle diseases that are listed in categories C, D and E. Slovenia has five control programmes (CPs) for the latter cattle diseases: bovine viral diarrhoea (BVD), infectious bovine rhinotracheitis (IBR), enzootic bovine leukosis (EBL), bluetongue and anthrax.

**Introduction, paragraph one, two and three:**

A list of 24 diseases listed under category C, D, or E of the new European Animal Health Law (AHL) [(EU) 2016/429] controlled in at least one member country has recently been compiled (2020) as part of the European Union (EU) COST action SOUND control (CA17110) (1).

In Slovenia there are five control programmes (CPs) in place for infectious cattle diseases with a lower categorization in the AHL (C, D, or E) and a directive for controlling *Salmonella* spp. outbreaks on farms. The CPs are designed to take account of the specific cattle rearing situation in Slovenia (communal alpine pastures, lack of fattening calves, the close proximity of farms, and small herds) and the geographical conditions.

This paper reviews the structure of the Slovenian cattle industry, the details of the existing CPs and provides the status for the other cattle diseases in categories C, D, or E of the AHL for which CPs are in place within Europe.

**FACTORS AFFECTING NON-REGULATED DISEASE CONTROL subtitle:**

“FACTORS AFFECTING CATTLE DISEASE CONTROL IN SLOVENIA”

**FACTORS AFFECTING NON-REGULATED DISEASE CONTROL, paragraph one:**

In Slovenia, the supply of beef calves does not meet demand. Therefore, farmers import calves from Middle and Eastern European countries. In the period between 2010 and 2016, most of the calves were imported from the Czech Republic (58.2%), followed by Hungary (10.2%), Romania (9.5%), and Slovakia (9.2%) (9). Imported calves are usually cheaper than those originating from Slovenia. The health status of imported calves is not checked for diseases in categories C, D, or E of the AHL and quarantine is not carried out before they are introduced into the herds, as it is not mandatory.

**Description of Existing Control Programmes in Slovenia for Non-Eu Regulated Cattle Diseases subtitle:**

“DESCRIPTION OF EXISTING CONTROL PROGRAMMES IN SLOVENIA FOR SELECTED CATTLE DISEASES”

**Epidemiological Situation for Other Eu Non-regulated Diseases subtitle:**

“Epidemiological Situation for Other Cattle Diseases Listed Under Category C, D, or E in the Animal Health Law”

**Epidemiological Situation for Other Eu Non-regulated Diseases, paragraph one:**

A COST action SOUND control is researching the cattle diseases listed under category C, D, or E in the AHL for which CPs exist in European countries. The action has compiled a list of 24 diseases that are controlled in at least one country (1). The Slovenian status for these diseases not already mentioned in the text is shown in (Table 2).

**Table 2 caption:**

“Disease status for Slovenia of cattle diseases listed under category C, D, or E in the Animal Health Law for which CPs exist in European countries.”

**Discussion, paragraph six:**

Regarding the number of cattle diseases listed under category C, D, or E in the AHL (investigated by SOUND control) controlled in European countries, Slovenia is below average with five CPs. The average in Europe is eight CPs per country. Diseases controlled in Slovenia are also controlled in most other European countries (1). The disease status for the controlled diseases is similar to the statuses of other countries in the region, with Austria having a favourable status for BVD and IBR, and Italy having regions free of IBR or regions with compulsory CPs (49). The country's status for the diseases that are not controlled in Slovenia (Table 2) are similar to the statuses of the neighbouring countries (46).

**Conclusion:**

Slovenia has five CPs in force for cattle diseases listed under category C, D, or E in the AHL for which CPs exist in European countries, which are the result of the specific cattle rearing conditions in the country and the wider region. The goal is to achieve eradication or control of all these diseases and add additional CPs for other diseases, which would increase the commercial value of Slovenian cattle, improve production, and animal welfare.

The authors apologize for these errors and state that this does not change the scientific conclusions of the article in any way. The original article has been updated.

## Publisher's Note

All claims expressed in this article are solely those of the authors and do not necessarily represent those of their affiliated organizations, or those of the publisher, the editors and the reviewers. Any product that may be evaluated in this article, or claim that may be made by its manufacturer, is not guaranteed or endorsed by the publisher.

